# Use of Chitosan-PVA Hydrogels with Copper Nanoparticles to Improve the Growth of Grafted Watermelon

**DOI:** 10.3390/molecules22071031

**Published:** 2017-06-22

**Authors:** Homero González Gómez, Francisca Ramírez Godina, Hortensia Ortega Ortiz, Adalberto Benavides Mendoza, Valentín Robledo Torres, Marcelino Cabrera De la Fuente

**Affiliations:** 1 Departamento de Horticultura, Universidad Autónoma Agraria Antonio Narro, Saltillo 25315, Coahuila, Mexico; homer_1447hotmail.com (H.G.G.); godramf@gmail.com (F.R.G.); abenmen@gmail.com (A.B.M.); varoto@prodigy.net.mx (V.R.T.); 2 Centro de Investigación en Química Aplicada, Blvd. Enrique Reyna Hermosillo No. 140, Saltillo 25294, Coahuila, Mexico; hortensia.ortega@ciqa.edu.mx

**Keywords:** graft, morphology, copper nanoparticles, productivity, chitosan-PVA hydrogels, watermelon

## Abstract

Modern agriculture requires alternative practices that improve crop growth without negatively affecting the environment, as resources such as water and arable land grow scarcer while the human population continues to increase. Grafting is a cultivation technique that allows the plant to be more efficient in its utilization of water and nutrients, while nanoscale material engineering provides the opportunity to use much smaller quantities of consumables compared to conventional systems but with similar or superior effects. On those grounds, we evaluated the effects of chitosan-polyvinyl alcohol hydrogel with absorbed copper nanoparticles (Cs-PVA-nCu) on leaf morphology and plant growth when applied to grafted watermelon cultivar ‘Jubilee’ plants. Stomatal density (SD), stomatal index (SI), stoma length (SL), and width (SW) were evaluated. The primary stem and root length, the stem diameter, specific leaf area, and fresh and dry weights were also recorded. Our results demonstrate that grafting induces modifications to leaf micromorphology that favorably affect plant growth, with grafted plants showing better vegetative growth in spite of their lower SD and SI values. Application of Cs-PVA-nCu was found to increase stoma width, primary stem length, and root length by 7%, 8% and 14%, respectively. These techniques modestly improve plant development and growth.

## 1. Introduction

Watermelon (*Citrulluslanatus* var. *lanatus*) is an Old World agricultural species, originally domesticated in Africa and from there spread throughout the Mediterranean, Middle East, and India. It was first established in the north of Mexico, although there is a possibility that it was introduced by African slaves brought to Mexico by the Spanish [1]. Watermelon has garnered much interest due to its high concentrations of compounds with antioxidant activity [2]. Mexico is one of the principal producers and exporters of watermelon, along with China, Turkey, and the United States [3]. Figures compiled in the FAOSTAT database indicate that Mexico produced close to a million tons of watermelon in 2014 [4].

In the ongoing effort to improve the quality and quantity of agricultural production, much work has been done with grafting, a technique whereby the living tissue from two different plants are joined and begin to develop as a single plant [5]. The technique originated in Asia, specifically Japan, where it has been used since 1920 [6]. Its use with the Cucurbitacae has had favorable results, allowing the introduction of cultivars in unfavorable areas or under unfavorable circumstances, as well as increasing the yields and quality of the fruit [7]. There are many grafting techniques used with watermelon, but tongue approach grafting results in a suitable rate of post-graft survival and adequate development of grafted plants [8].

Apart from grafting, the use of chitosan (Cs)-a derivative of the chitin found in the carapaces of crustaceans, mollusks, insects, and the cell walls of fungi-has been studied [9,10,11,12]. It is widely used thanks to its properties of non-toxicity, biocompatibility, and biodegradability [13]. It is capable of forming hydrogels when combined with polyvinyl alcohol (PVA) [14]. Hydrogels are characterized as insoluble three-dimensional structures with the ability to absorb water [15]. Hydrogels made from Cs-PVA are hydrophilic, non-toxic and biocompatible, possess ideal mechanical properties, and are stable for long periods across a range of temperatures and pHs [16]. These properties make Cs-PVA hydrogels an ideal media for the controlled release of nanomaterials, such as copper nanoparticles [17].

Nanoparticles (NPs) are clusters of atoms, ranging from 1–100 nm in diameter, that behave as a single unit. Copper NPs have a diameter of between 5–25 nm, although this depends on the temperature and pressure used during their manufacture [18]. In agriculture, copper NPs are used as a treatment for some plant diseases and as an adjuvant for essential nutrient assimilation, resulting in the reduced use of agricultural consumables compared to traditional cultivation strategies [19].

The advent of nanotechnology has been one of the most significant modern technological advancements, allowing the production and utilization of new materials manufactured at scales where they demonstrate novel effects and properties as compared to those seen at conventional scales [20]. Nanoparticles, in particular, have demonstrated better durability, increased chemical reactivity, and greater efficiency in biomedical, pharmaceutical, electronic-and more recently-agricultural applications [21]. Nanotechnology has the potential to revolutionize our approach to human health. Its positive effects on the durability of certain materials have been well documented across different fields [22]. However, the effects these novel nanomaterials could have on grafted crops and the impact of micro-morphological modifications on productivity are not well known. Here we report the changes in plant growth and stomatal morphology of grafted ‘Jubilee’ watermelon cultivated with Cs-PVA-nCu hydrogels.

## 2. Results and Discussion

### 2.1. Plant Growth Analysis

The effects of grafting on the length of the watermelon primary stem and stem diameter are presented in Table 1. A statistically significant difference between treatments was observed for both variables, with stem length increasing 20% and stem diameter increasing by 17%. These values are similar to previously reported results from a study where grafted watermelon plants demonstrated superior vegetative growth [23]. It is argued that grafted plants grow and develop better due to an increased capacity for nutrient and water acquisition [24]. The increases in the growth of stems and the trailing vines of grafted watermelon plants have been found to have effects on the overall vegetative growth and fruit production [25].

The application of Cs-PVA-nCu also had a significant effect on the stem length, demonstrating an 8% increase as compared to the untreated control. No significant changes were observed in the diameter of the stems (Table 2). It is thought that the use of nanomaterials may stimulate vegetative growth in some species by inducing antioxidant activity within the plant, as well as potentially acting as elicitors, which would also favor vigorous development and growth [26]. Treatment with only Cs-PVA hydrogels also resulted in a significant 7% increase in stem length, as compared to the untreated control. Chitosan applied on its own has been found to have positive effects on the development and growth of some crops. Its application stimulates the plant’s defense mechanisms that in turn promote vegetative growth, although the precise relationship between plant defense inducers and primary metabolism is not known [27].

Grafting had a significant effect on the production of plant biomass as measured 60 days after transplanting (Table 3). Grafted watermelon plants demonstrated increases in fresh weight (25%), dry weight (7%), root length (15%), and specific leaf area (39%) as compared to non-grafted plants. It is evident that grafted plants have a more robust character than their non-grafted counterparts do, thanks to the vigor conferred by the rootstock [28]. A similar observation was made for cucumbers, where greater production of vegetative and radicular biomass was obtained with the use of grafts [29].

In Table 4, the effects of Cs-PVA-nCu application on plant biomass production are presented. Treatment with the hydrogels containing copper NPs favored radicular development (14% increase) but did not have any effect on plant fresh weight, dry weight, and specific leaf area. The positive effect on root growth may be due to the copper NPs affecting the efficiency of plant primary metabolism [30]. Although, it should be noted that the Cs-PVA hydrogels also had a significant effect on root biomass and that the presence of chitosan has been found to have favorable effects [31].

In Table 5, the combined effect of the graft and the use of hydrogels are presented where it was found that for the fresh weigh and dry weight variables, the interaction of the factors seems to have no effect since no significant difference was found when combining them, but if for the stem length variable where the best the best results were grafted plants also the stem diameter and specific leaf area were favored when using plants grafted with and without copper NPs in chitosan hydrogels in other hand the best treatment for root length was grafted plants and Cs hydrogel. In general, it is observed that the grafted plants improve the response for most of the variables evaluated.

### 2.2. Micro-Morphology Analysis

**Figure 1:**
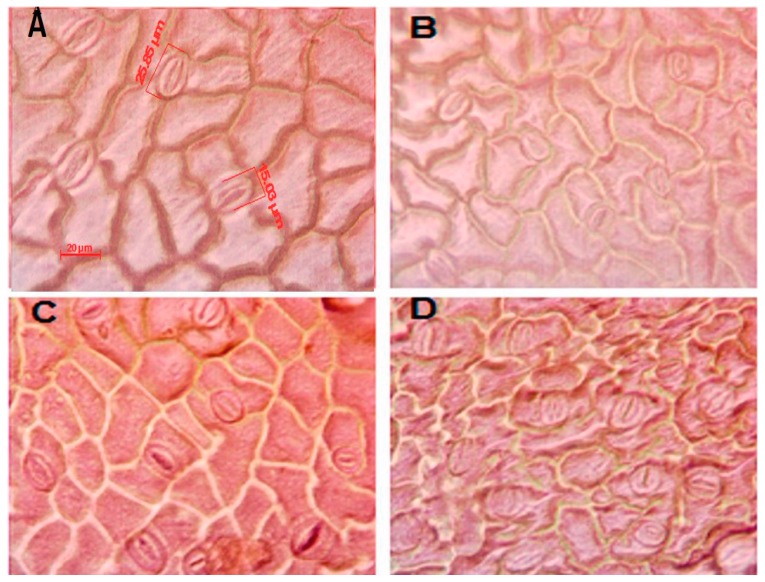
Distribution and size of stomata. The adaxial leaf surface with (**A**) and without (**B**) grafting. The abaxial side of leaves from grafted (**C**) and non-grafted (**D**) plants treated with Cs-PVA-nCu. Scale bar, 20 µm.

With respect to the micro-morphological characteristics considered in our study, significant differences between the grafted and non-grafted watermelon plants were observed (Table 6). Adaxial and abaxial SD was reduced 18% and 9%, respectively, in grafted plants. Adaxial SI in grafted plants was also reduced by 19% but showed no significant change on the abaxial side (Figure 1). No significant changes were seen in the other variables, SL and SW. These results are in line with a previously published study evaluating the effects of grafting on the SD and SI of cucumber, observing a reduction in both variables and characterizing it as an effect of the grafts [32].

Treatment with Cs-PVA-nCu appears to have minor effects on the plant micro-morphological variables measured (Table 7). No significant changes to adaxial and abaxial SD and SI were observed among different treatments. The only evident change recorded was a 7% increase in the width of adaxial stomata in the leaves of plants exposed to the copper NPs. This variation could be due to stress response genes activated in the presence of copper exerting downstream effects on biochemical and physiological processes [33]. We believe the elicited plant response could have been stronger if a higher experimental concentration of copper NPs had been used [34].

The response of the variables to the combination of factors is presented in (Table 8) where the best treatments are plants without grafting with application of Cs-PVA hydrogel as well as plants without grafting and without any application since they registered the highest values for DE and IE, this response was only observed in the adaxial zone of the leaves. Additionally, the SI-Cs-PVA treatment improves the stoma width by 5%.

## 3. Materials and Methods

### 3.1. Experiment Location

The present work was undertaken in the experimental field belonging to the Department of Horticulture of the Autonomous Agrarian University Antonio Narro. The plot is located in Buenavista, Saltillo, Coahuila, Mexico, at latitude 25°21’22.51”, longitude 101°2’9.88” and 1760 meters above sea level.

### 3.2. Plant Material and Growth Conditions

‘Jubilee’ watermelon scions were grown from seed. This watermelon cultivar is characterized by large, elongated fruit with brilliant red pulp and a striped rind pattern [35]. A squash hybrid (Cucurbita maxima x Cucurbita moschata) was chosen for the rootstock, as it is greatly amenable to cucurbitaceous grafts [36]. This rootstock also stimulates vigorous growth under adverse climate and soil conditions, thanks to a larger root system that allows more efficient water and nutrient assimilation [37].

The watermelon seeds were sown on 31 May 2016 in 60-cell polystyrene seed trays filled with peat moss, one seed per cell. Eight days later, the rootstock seeds were sown in 128-cell seed trays filled with peat moss, one seed per cell. The rootstock was sown later to account for its greater vegetative vigor and ensure the rootstock and scion stems would be of similar sizes.

### 3.3. Cs-PVA Hydrogel Synthesis and Copper NP Absorption

Chitosan (200,000 M.Wt) was purchased from Marine Chemical (Kerala, India). The polyvinyl alcohol (30,000–50,000 M.Wt, 98% hydrolyzed) was purchased from Sigma-Aldrich (St. Louis, MO, USA). Copper nanoparticles (99.8%, 25 nm) were obtained from SkySpring Nanomaterials, Inc. (Houston, TX, USA).

The Cs-PVA hydrogels were synthesized in the Applied Chemistry Research Center (Saltillo, Coahuila, Mexico) pilot plant. First, 2% (*w/v*) chitosan (250 mL) and 4% (*w/v*) polyvinyl alcohol (250 mL) were mixed for two hours at 300 rpm and 60 °C to obtain a hydrogel with a 1:2 relation of chitosan to PVA. Then, 50% glutaraldehyde (2.27 mL) was added as the cross-linking agent, and the solution stirred for five minutes at 450 rpm and 25 °C. Finally, 6% (*w/v*) NaOH (100 mL) was added and the solution stirred for a further hour at 300 rpm and 25 °C. The Cs-PVA hydrogels were immediately collected and washed with distilled water and ethanol. The hydrogels were allowed to dry and weighed [38].

The copper NPs (100 mg) were dispersed in a 1% (*w/v*) Tween solution by sonication (50 watts, 70% frequency) for five minutes. Dilutions were prepared to obtain a final concentration of 0.4 mg copper NPs. The Cs-PVA hydrogels (1 g) were allowed to soak in the dilute NP solution and then dried at 60 °C.

### 3.4. Grafting and Application of Cs-PVA-nCu Hydrogels

Grafting was performed on 15 July 2016, two weeks after the sowing of the watermelon scions and five days after the emergence of the squash rootstock. Scion and rootstock were grafted according to the tongue approach method described in Oda et al. [39] and returned to seed trays. The grafted plants were kept under greenhouse conditions with an average temperature of 30 °C and 60% relative humidity. The plants were watered daily to prevent wilting.

After 15 days following grafting, the watermelon stems were cut below the graft site and the vegetative growth from the rootstock was removed. The grafted plants were kept a further seven days under the same greenhouse conditions.

After successful anastomosis at the graft site, the plants were moved out of the greenhouse and placed under shade netting. They were transplanted into 10 L pots, containing a 1:2 mix of peat moss to perlite. The Cs-PVA-nCu hydrogels were applied by dividing 1 g of hydrogel containing 0.4 mg of copper NPs into thirds (~0.33 g) and distributing them equally throughout the potting mix as the pots were filled by thirds as well. Cs-PVA hydrogels without the copper NPs were applied in the same manner.

The experimental design was randomized and each experimental unit consisted of four plants. The treatments were defined by a factorial arrangement of two graft states (grafted or not grafted plants) and three levels of hydrogel application (plants treated with Cs-PVA-nCu hydrogel, only Cs-PVA hydrogel, or no application at all). The six treatments were repeated three times.

### 3.5. Measurement of Plant Growth Variables

The primary stem length, the stem diameter, the specific leaf area, root length, and both fresh and dry weights of the vegetative foliage were monitored as indicators of growth. The primary stem length and stem diameter were measured weekly, selecting four plants per treatment in a random fashion and beginning eight days after transplanting. To obtain values for fresh and dry weight, root length, and specific leaf area after a growth period of 60 days, four plants per treatment were selected and consumed in the sampling. Leaf area was measured using a LI-COR 3004 imaging system and final specific leaf area values given in cm^2^·g^−1^ dry weight.

### 3.6. Measurement of Micro-Morphological Variables

The stomatal density (SD), length (SL), and width (SW) of the adaxial and abaxial stomata and a calculated stomatal index (SI) were measured. Four plants per treatment were selected, from which two adult leaves were taken from the primary stem. Ensuring each leaf was oriented the same way, an impression of the epidermis was made according to the micro-relief method [40]. The impressions were observed under a VWR VistaVision compound microscope (Radnor, PA, USA) with an integrated Pixera (Chicago, IL, USA) PVC 100C camera using the 10× objective. Digital images were captured and later analyzed with the AxioVision rel. 4.8 image processing software (Carl Ziess AG, Oberkochen, Germany).

The leaf area observed in each field was found to measure 0.0247 mm^2^. For each sample, three images were randomly selected and the number of stomata and epidermal cells was counted. The stomatal index (SI) was calculated with the formula [41].

(1) $$\mathrm{SI}=100\times \left[\frac{\mathrm{NS}}{\mathrm{EC}+\mathrm{NS}}\right]$$

where NS is the number of stomata and EC is the number of epidermal cells. The stomatal density was calculated as

(2) $$\mathrm{SD}=\frac{\mathrm{Number}\;\mathrm{of}\;\mathrm{stomata}\;\mathrm{per}\;\mathrm{field}}{\mathrm{area}\;\mathrm{of}\;\mathrm{the}\;\mathrm{visualized}\;\mathrm{field}}$$

### 3.7. Data Analysis

The Statistical Analysis System (SAS) version 9.4 software package was used to analyze the obtained data. The results from the treatments were subjected to Tukey’s test (α ≤ 0.05) in order to determine if any were statistically different.

## 4. Conclusions

Subjecting watermelon plants to grafting modifies the leaf micromorphology, inducing a reduction in stomata-associated variables but stimulating increases in the parameters associated with biomass production. This suggests that changes in the plant stomata induced by grafting have a favorable impact on plant development and growth. Grafting confers an indubitable vigor to the plants, perhaps improving their ability to take up water and nutrients from their environment, which translates into a superior growth rate. Similarly, the application of Cs-PVA-nCu and copper-deficient Cs-PVA hydrogels stimulated the growth of the primary stems, the root system, and an increase in stomatal width, even though the concentration of copper NPs used was low. This leaves the topic open to further investigations geared towards developing new technological applications that will complement the eco-friendly agricultural technologies already in use.

## Figures and Tables

**Table 1:** Effect of grafting on watermelon growth variables.

Treatment	Stem Length (m)	Stem Diameter (mm)
Grafted	3.08 a	11.25 a
Non-grafted	2.472 b	9.43 b

Note: Values with different alphabets are significantly different (Tukey α ≤ 0.05).

**Table 2:** Effect of Cs hydrogel treatments on selected watermelon growth variables.

Treatment	Stem Length (m)	Stem Diameter (mm)
Cs-PVA-nCu	2.85 a	10.77 a
Cs-PVA hydrogel	2.82 a	10.18 a
No treatment	2.65 b	10.06 a

Note: Values with different alphabets are significantly different (Tukey α ≤ 0.05).

**Table 3:** Effect of grafting on watermelon biomass production.

Treatment	Fresh Weight (g)	Dry Weight (g)	Root Length (cm)	Specific Leaf Area (cm^2^·g^−1^)
Grafted	75.72 a	5.43 a	30.67 a	206.60 a
Non-grafted	56.95 b	5.13 b	25.92 b	127.00 b

Note: Values with different alphabets are significantly different (Tukey α ≤ 0.05).

**Table 4:** Effect of Cs hydrogel treatments on watermelon biomass production.

Treatment	Fresh Weight (g)	Dry Weight (g)	Root Length (cm)	Specific Leaf Area (cm^2^·g^−1^)
Cs-PVA-nCu	70.16 a	5.36 a	30.00 a	174.34 a
Cs-PVA	65.00 a	5.35 a	29.00 ab	167.32 a
No Treatment	63.83 a	5.13 a	25.87 b	158.80 a

Note: Values with different alphabets are significantly different (Tukey α ≤ 0.05).

**Table 5:** Combined effect of grafting and application of gels on watermelon growth a biomass production.

Treatment	Stem Length (m)	Stem Diameter (mm)	Fresh Weight (g)	Dry Weight (g)	Root Length (cm)	Specific Leaf Area (cm^2^·g^1^)
CI-Cs-PVA-nCu	3.11 a	12.00 a	72.33 a	5.56 a	31.00 ab	221.22 a
CI-Cs-PVA	3.13 a	10.75 ab	79.50 a	5.57 a	32.75 a	195.63 ab
CI-No Treatment	3.00 a	11.00 a	75.33 a	5.18 a	28.25 ab	203.10 a
SI-Cs-PVA-nCu	2.590 b	9.55 bc	57.68 a	5.14 a	27.00 ab	127.46 c
SI-Cs-PVA	2.52 bc	9.62 bc	60.83 a	5.16 a	27.25 ab	139.01 bc
SI-No Treatment	2.30 c	9.12 c	53.33 a	5.09 a	23.50 b	114.50 c

Note: Values with different alphabets are significantly different (Tukey α ≤ 0.05); CI = grafted, SI = non grafted.

**Table 6:** Effect of grafting on watermelon leaf micromorphology.

Treatment	Adaxial				Abaxial			
	SD	SI	SL	SW	SD	SI	SL	SW
Grafted	228.5 b	17.0 b	23.6 a	13.27 a	242 b	30.42 a	21.54 a	13.26 a
Non-grafted	278.9 a	20.8 a	22.9 a	13.34 a	265 a	28.97 a	21.13 a	13.50 a

Note: Values with different alphabets are significantly different (Tukey α ≤ 0.05).

**Table 7:** Effect of Cs hydrogel treatments on watermelon leaf micromorphology.

Treatment	Adaxial				Abaxial			
	SD	SI	SL	SW	SD	SI	SL	SW
Cs-PVA-nCu	262.18 a	19.48 a	23.70 a	13.83 a	250.42 a	28.28 a	21.67 a	13.40 a
Cs-PVA	257.77 a	19.09 a	23.12 a	13.13 ab	252.10 a	29.84 a	21.21 a	13.25 a
No Treatment	245.37 a	17.99 a	22.90 a	12.94 b	258.82 a	30.97 a	21.06 a	13.49 a

**N**ote: Values with different alphabets are significantly different (Tukey α ≤ 0.05).

**Table 8:** Combined effect of grafting and application of gels on watermelon leaf micromorphology.

Treatment	Adaxial				Abaxial			
	SD	SI	SL	SW	SD	SI	SL	SW
CI-Cs-PVA-nCu	222.85 b	16.18 c	24.18 a	12.82 ab	242 a	28.20 a	21.37 a	13.65 a
CI-Cs-PVA	225.21 b	17.19 bc	22.27 a	13.47 ab	242 a	31.20 a	21.14 a	12.97 a
CI-No Treatment	238.65 ab	17.51 bc	24.07 a	13.49 ab	242 a	31.85 a	20.68 a	13.16 a
SI-Cs-PVA-nCu	268.90 ab	19.79 ab	23.22 a	13.05 ab	258 a	28.35 a	21.98 a	13.15 a
SI-Cs-PVA	282.35 a	21.00 a	23.78 a	14.19 a	262 a	28.47 a	21.28 a	13.53 a
SI-No Treatment	285.71 a	21.45 a	21.73 a	12.78 b	275 a	30.10 a	21.25 a	13.83 a

Note: Values with different alphabets are significantly different (Tukey α ≤ 0.05). CI = grafted, SI = non grafted.
